# (De)Constructing invisible barriers: the gender projection model of organizational inequality

**DOI:** 10.3389/fsoc.2025.1686983

**Published:** 2025-11-19

**Authors:** Fabrice Gabarrot

**Affiliations:** 1 Laboratoire Psy-Drepi, Université Bourgogne Europe, Dijon, France; 2 Unidistance, Brig, Switzerland

**Keywords:** gender inequality, organizational prototypes, Ingroup projection, Workplace discrimination, social identity theory, glass ceiling

## Abstract

Gender inequality in contemporary organizations persists despite decades of policy initiatives, partly because many barriers have shifted from overt exclusion to subtle, often invisible, mechanisms embedded in everyday practices. Existing models—whether grounded in economics, sociology, or social psychology—tend to focus on either the “supply” of candidates or the “demand” of organizations, reify gender categories, and overlook the active role of dominant groups in defining competence standards. This article introduces the Gender Projection Model (GPM), an identity–structural framework that explains how dominant-group members project their own attributes, life patterns, and interactional styles onto the prototypes of valued organizational roles such as leaders, experts, or the “ideal worker.” These prototypes, presented as neutral, are in fact historically situated and power-sensitive, shaping both evaluation criteria and the aspirations of those perceived as non-prototypical. The GPM predicts that projection is strongest when the gender hierarchy is perceived as legitimate, stable, and impermeable, and that it operates as a feedback loop: prototypes influence evaluations and opportunities, which in turn reinforce status beliefs and prototype stability. By reframing “supply” as a product of organizational demand, the model unifies phenomena often treated separately—glass ceiling, sticky floor, glass cliff, backlash, tokenism—within a single identity-driven mechanism. Beyond its theoretical integration, the model generates testable predictions about when projection strengthens or weakens and offers an empirical and diagnostic framework for organizational analysis. This article thus outlines testable implications, proposes a cumulative research agenda, and discusses practical and organizational interventions aimed at redefining prototypes to foster equitable access to valued roles.

## 1 Introduction

Gender discrimination in the workplace, far from being a residual issue, remains a central challenge for contemporary sociology and organizational research. The “quiet revolution” (Goldin, 2006) appears to have plateaued, as evidenced by the stagnation of key equality indicators since the late twentieth century (Blau, 2024; England et al., 2020). This stagnation manifests as a constellation of both visible and invisible barriers. The glass ceiling slows women's access to the highest echelons of power. The sticky floor keeps them in the least valued positions. More diffuse barriers operate continuously across organizational routines. They range from biased evaluations and homophilous networks to implicit criteria of competence rooted in the “ideal worker” norm—a dynamic powerfully illustrated by empirical research on the motherhood penalty and devotion schemas (Correll et al., 2007; Williams, 2013) and by recent evidence that fields emphasizing innate brilliance show lower female representation (Leslie et al., 2015). Yet even when women manage to cross these thresholds, they are often appointed to precarious or highly exposed positions (i.e., glass cliff positions; Ryan et al., 2011) and penalized for enacting the very behaviors associated with leadership (backlash; Rudman et al., 2012).

Dominant frameworks share two blind spots that signal a paradigmatic impasse. First, they reify gender by treating it as a set of fixed traits or historically self-perpetuating hierarchies rather than as a dynamic relation embedded in power and practice. Theories such as role congruity or lack-of-fit often assume shared and static stereotypes, while sociological accounts sometimes portray inequality as a legacy that reproduces itself almost automatically. In both cases, gender appears as a property rather than a process.

Second, these approaches, whether economic, sociological, or psychological, focus primarily on the constraints of the dominated group—women's preferences, motivations, or structural disadvantages—while neglecting the mechanisms of dominance that actively sustain inequality. By centering explanation on those constrained rather than those who constrain, they obscure the driver of social reproduction: the situated, identity-motivated practices through which members of dominant groups define and police the standards of competence that preserve their advantage (Kalev and Deutsch, 2018).

To theorize this missing mechanism, we introduce the Gender Projection Model (GPM). The GPM explains how members of dominant groups project their own attributes, life patterns, and interactional styles onto the prototypes of valued organizational roles—leaders, experts, or the so-called “ideal worker.” These prototypes, presented as neutral or meritocratic, are in fact historically situated and power-sensitive: they embody the norms and life conditions of those who occupy positions of dominance. Once institutionalized, these dominant-anchored standards shape both the evaluative criteria used by organizations and the aspirations of those who perceive themselves as non-prototypical. The result is a feedback process through which inequality is continually reproduced, not merely through bias or exclusion, but through the very definitions of competence and professionalism that govern access to valued roles.

Crucially, while our account foregrounds prototype construction and maintenance by dominant actors (the demand side), the GPM is two-sided, explaining how these prototypes in turn manufacture “supply” by shaping the aspirations and self-selection of non-prototypical members. Although the present article focuses on gender as a paradigmatic case, the projection mechanism is structurally generalizable and can, in principle, extend to other bases of inequality such as race, class, or parental status.

To build our argument, we draw a key analytical distinction between prototypes and stereotypes. Prototypes define what counts as a good instance of a valued role (e.g., a good leader), while stereotypes describe social groups (e.g., women are communal). The GPM posits that dominant groups project the latter onto the former, transforming descriptive gender stereotypes into normative professional standards. In this view, non-prototypicality reflects the perceived distance between a candidate and the operative prototype of a role. Perceived legitimacy, stability, and permeability of the hierarchy act as socio-structural beliefs that moderate the strength of projection.

*Scope and contribution*. The GPM integrates phenomena often treated separately—the glass ceiling, sticky floor, glass cliff, backlash, and the maternal wall—within a single identity-driven mechanism of prototype construction. By reframing the classical supply–demand divide, it reconceptualizes “supply” not as an exogenous factor but as an outcome of the normative power of prototypes. The remainder of this article develops the model's theoretical foundations, specifies its testable implications, and outlines its methodological and organizational relevance.

## 2 Theoretical rationale: limitations of dominant approaches

### 2.1 The false supply–demand dichotomy

Debates about workplace gender inequality have long been structured around a persistent divide between *supply-side* and *demand-side* explanations (Stockdale and Nadler, 2013; Bridges and Nelson, 2018). The neoclassical economic paradigm privileges the former, attributing disparities to individual choices, stated preferences, or rational expectations derived from differences in human capital (Jaspers et al., 2022). In contrast, critical sociology emphasizes demand, showing how the structures, rules, and norms of gendered organizations (Acker, 1990) sustain both vertical and horizontal segregation (Kalev and Deutsch, 2018). Social psychology has often sought to bridge these perspectives by examining how stereotypes and role-based biases (Schein, 1973; Eagly and Karau, 2002) simultaneously shape the aspirations of actors and the evaluative judgments of decision-makers.

While this typology remains heuristically useful, it rests on an artificial separation that obscures the reciprocal shaping of supply and demand. Recent research demonstrates that so-called supply factors—for example, career aspirations or family choices—are themselves products of demand-side forces such as recruitment criteria, organizational cultures, and sponsorship practices (Fernandez-Mateo and Kaplan, 2018; Lluent et al., 2023). A woman's withdrawal from a leadership track, for instance, often reflects the cumulative effects of biased competence assessments, exclusionary networks, and cultures of constant availability that make participation an unsustainable cost (Fernandez-Mateo and Kaplan, 2018; Son Hing et al., 2023).

This mutual interdependence unfolds across multiple levels—societal, organizational, interpersonal, and individual—and tends to cumulate over time (Son Hing et al., 2023). The interaction between an occupation's gender composition, task interdependence, and shared beliefs about competence helps explain the persistence of wage gaps (Meuris and Elías, 2022). These dynamics are further conditioned by macro-contextual factors such as labor-market volatility or national institutional configurations (Jung et al., 2021; Nishitani and Kawaguchi, 2023).

Disciplinary boundaries have contributed to the endurance of this divide. Each field tends to privilege its own level of analysis—macro-structural in sociology, micro-behavioral in psychology, and meso-organizational in management studies—thereby overlooking frameworks capable of articulating these scales. Yet, as Doise (1986) argued, explaining social phenomena requires linking four analytically distinct but interdependent levels of explanation: intra-individual (cognitive and motivational processes), inter-individual (interactions and power relations), positional (status relations), and ideological (belief systems and cultural frames).

The Gender Projection Model (GPM) builds on this multi-level perspective by reframing what typically appears as a “supply-side choice” as *endogenous* to prototype governance. Signals of (mis)fit generated by demand-side structures shape the horizons of aspiration, the perceived costs of role performance, and the calculus of assimilation, resistance, or exit. However, even these integrative perspectives remain constrained by a deeper epistemic limitation: the reification of gender categories themselves—a limitation to which we now turn.

### 2.2 The common ground: the reification of gender categories

Beyond their methodological differences, both supply-side and demand-side approaches share a positivist ontology that takes gender as given, treating it as an empirical variable rather than a social relation. This reification, at once ontological and historical, transforms a relation of power into a variable of description. In most accounts, “men” and “women” are treated as natural, stable, and transhistorical entities rather than as relational and situated positions embedded within power relations. This dual naturalization—ontological and temporal—obscures the dynamic and contested nature of gender, flattening complexity and detaching analysis from the situated practices through which hierarchy is continuously reproduced.

Reification takes two closely related forms. The first, essentialism, assumes that stereotypes or “gender roles” reflect underlying psychological or biological realities—a “kernel of truth” (Klineberg, 1950). This stance naturalizes difference by portraying social disparities as stemming from intrinsic dispositions and concealing the legitimizing function of stereotypes: to stabilize and rationalize the social order (Hoffman and Hurst, 1990). Stereotypes thereby operate as instruments of power, recasting hierarchy as the natural expression of universal difference.

The second, historicism, explains persistence by turning history itself into a cause. Foundational work has rightly traced contemporary inequalities to the legacy of the Industrial Revolution and the ideology of “separate spheres” (Padavic and Reskin, 2002). Yet this genealogy becomes problematic when continuity is mistaken for determinism—when history is treated as inevitability rather than as a contingent and conflictual process. In such accounts, the present appears as a mere echo of the past, and the active mechanisms of reproduction remain unspecified.

Both forms of reification transform a conflictual social relation into a static descriptive difference, detaching it from the everyday interactions and organizational practices—rules, routines, criteria, and networks—through which hierarchy is reproduced. In privileging either the micro (attitudes and choices) or the macro (structures and legacies), they foreclose articulation across levels of explanation.

Social Role and Role Congruity theories occupy an intermediate space. Rooted in the gendered division of labor, they allow stereotypes to evolve with occupational roles (Eagly and Karau, 2002). Yet the recent revival of the “kernel of truth” thesis (Eagly and Hall, 2025) reasserts essentialism by treating group mean differences as empirical regularities and benchmarks of descriptive accuracy. Three concerns follow. First, the ecological fallacy: group means do not prescribe individual judgments, and distributions overlap widely. Second, endogeneity: observed “truths” often reflect criteria, evaluation formats, and job designs that are themselves gendered. Third, a blind spot around power: who defines competence prototypes, in whose interest, and under which institutional rules?

What remains missing, then, is a framework capable not only of resisting both essentialist and historicist reification, but of re-specifying how power and identity interact in the very construction of competence standards. The next section introduces such a framework: the Gender Projection Model (GPM)—which reframes reproduction itself as an identity-driven process of prototype construction.

### 2.3 Consequences: the missing motor of reproduction

These intertwined essentialist and historicist logics leave dominant models ill-equipped to explain phenomena in which motivational and contextual dynamics are pivotal—such as backlash (Rudman et al., 2012) or the glass cliff (Ryan et al., 2011). What remains absent is a psychosocial mechanism of reproduction that links identity work with organizational norms and practices. To address this gap, we introduce a model that specifies how valued roles such as “leader” are socially constructed through the interplay of cognitive, motivational, social, and organizational processes, and how contextual beliefs modulate these dynamics.

At the core of this missing mechanism lies the motivation to defend the gender hierarchy—a preconscious yet powerful force. In line with Ridgeway (2009), gender operates as a primary cultural frame that automatically activates status beliefs. The strength of this process stems from a fundamental human motive: the need to belong and to see one's ingroup valued (Baumeister and Leary, 1995). The apparent tension between identity-based motivation and normatively “rational” motives (e.g., ethics, performance) reflects an asymmetry between primary and secondary processes: ingroup favoritism emerges spontaneously, whereas egalitarian norms require effortful regulation (Falomir-Pichastor et al., 2011; Gabarrot and Falomir-Pichastor, 2017; Falomir-Pichastor et al., 2009).

In short, prevailing frameworks describe the patterns and domains of bias but not the underlying processes that sustain them. They lack an account of how the motivation to uphold a hierarchy translates into the everyday practices—evaluations, interactions, and norms—that reproduce inequality. The next section introduces a model designed to provide precisely this explanatory bridge.

## 3 Core proposition: the gender projection model

The critique of dominant paradigms on gender inequality reveals a central gap: the lack of a unifying mechanism explaining how gender hierarchies persist, reconfigure, and endure despite equality policies. Supply- and demand-side approaches capture key aspects of reproduction, yet they fail to integrate the dynamic interplay between identity-based motivations and the organizational structures that both express and reinforce them.

The Gender Projection Model (GPM) (Carrel et al., 2022) fills this gap by conceptualizing gender inequality as the product of an identity-driven, context-sensitive process in which the attributes and interactional styles of dominant-group members are projected onto the prototypes of valued roles. Through organizational structures and evaluative practices, these projections are institutionalized and come to define seemingly neutral standards of competence and legitimacy.

### 3.1 The identity-based foundation: the social identity approach and socio-structural beliefs

The Gender Projection Model (GPM) builds on the social identity approach, which integrates insights from Social Identity Theory (SIT; Tajfel, 1978; Tajfel and Turner, 1979) and Self-Categorization Theory (SCT; Turner et al., 1987). As Haslam (2012) emphasizes, this framework breaks with methodological individualism: group membership is a psychologically transformative process through which the self is defined in terms of “we” rather than “I.” This transformation reshapes motivation, cognition, and behavior, enabling cooperation, collective action, and the very formation of organizational cultures.

A central contribution of SIT lies in its analysis of socio-structural beliefs that regulate intergroup dynamics—specifically, the perceived *permeability* of group boundaries, the *legitimacy* of hierarchy, and its *stability* over time. When hierarchy is perceived as legitimate and stable, and boundaries as impermeable, dominated-group members are less likely to pursue mobility and more likely to internalize existing inequalities. Conversely, perceived illegitimacy, instability, or permeability invite collective challenge. This identity-based logic explains contextual variations that static stereotype or role-congruity models struggle to capture.

The social identity framework thus directly addresses the limits of essentialist and historicist accounts of inequality. Where essentialism naturalizes difference by attributing hierarchy to intrinsic qualities, the social identity approach demonstrates that hierarchy is sustained by socially constructed—and therefore reversible—beliefs. Where historicism portrays male dominance as an inherited constant, it highlights the conditions under which this legacy is reaffirmed or contested by actors themselves. In contrast with role congruity and status-based theories that assume shared cultural templates of competence, the GPM treats prototypes as contingent upon the perceiver's identity and the prevailing socio-structural beliefs. This shift yields distinct, falsifiable predictions: if prototype content varies systematically with evaluators' group membership and identification, then the assumption of a “shared template” is undermined. Under conditions of high legitimacy and stability, for instance, evaluators may penalize non-dominant candidates even when they conform to dominant prototypes (backlash), whereas instability or illegitimacy can instead generate “glass-cliff” openings.

These socio-structural beliefs also resonate with classic sociological concepts. *Legitimacy* echoes Weber's notion of legal-rational domination (Weber, 1978) and Bourdieu's (1977) concept of *doxa*; *stability* refers to the consolidation of relations through institutional routines and cultural norms; and *permeability* reflects structural opportunities for mobility. In organizational contexts, such beliefs shape how actors interpret their own position and possibilities. In settings perceived as legitimate and closed, women may internalize the belief that leadership roles are “not for them”—not through incapacity, but because recurrent organizational signals of exclusion constrain and redirect their aspirations. Conversely, when legitimacy or stability wavers—such as during crises—opportunities for contestation and non-traditional appointments tend to emerge.

**Figure 1:**
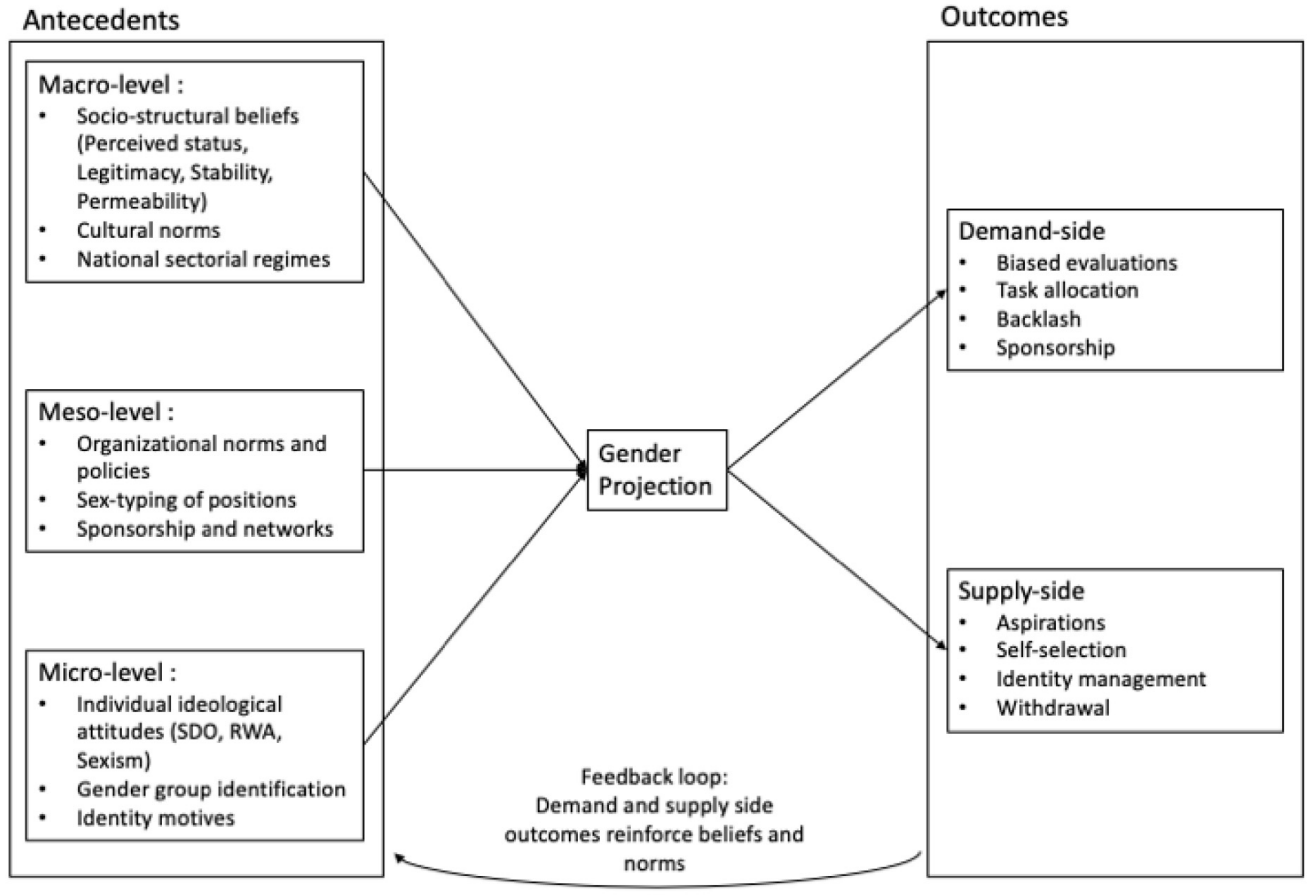
The Gender Projection Model (GPM). Antecedents at the macro (socio-structural beliefs, cultural norms, national regimes), meso (organizational norms, sex-typing of positions, networks), and micro levels (ideological attitudes, gender identification, identity motives) shape the process of gender projection, through which gender-group attributes are mapped onto valued role prototypes. These prototypes in turn generate demand-side outcomes (biased evaluations, task allocation, backlash, sponsorship) and supply-side outcomes (aspirations, self-selection, identity management, withdrawal). Through a feedback loop, these outcomes reinforce the antecedent beliefs and norms, thereby reproducing inequality.

In short, the social identity approach provides a dynamic model of both reproduction and transformation. Building on this foundation, the GPM conceptualizes how dominant-group members project their own attributes onto valued organizational roles, and how this projection is supported or undermined by the socio-structural beliefs that regulate perceptions of hierarchy. These moderators constitute a core dimension of the model, presented schematically in Figure 1 and comparatively in Table 1.

**Table 1:** A comparative analysis of frameworks on workplace gender inequality.

**Framework**	**Core assumption**	**What it explains well**	**Limitations**	**What GPM adds**
Supply-side (economics)	Disparities reflect individual choices, preferences, human capital	Opting out, career interruptions	Underestimates structural constraints; naturalizes preferences	Shows how “choices” are shaped by prototypes and status beliefs Reframes preferences as outcomes rather than givens
Demand-side (sociology)	Inequality reflects organizational rules, gendered structures	Segregation, barriers to promotion	Lacks account of agency and motivation	Explains how dominant actors actively project norms into roles Integrates agency and motivation while retaining structural insights
Role Congruity/Status accounts (social psychology)	Stereotypes as shared templates of competence	Backlash, lack of fit, double binds	Treats stereotypes as static; reifies categories; Tends to overlook meso-level dynamics	Prototypes are perceiver-contingent, contested, and moderated by socio-structural beliefs Shifts the unit of analysis from stereotypes of groups to prototypes of roles
GPM	Prototypes as identity-driven constructions by dominant groups	Unifies glass ceiling, sticky floor, glass cliff, backlash	–	Provides the motor of reproduction: identity-driven projection embedded in organizational practices and reinforced through feedback loops

### 3.2 The core mechanism: identity projection as the motor of reproduction

The Gender Projection Model (GPM) extends the social identity approach through the Ingroup Projection Model (Mummendey and Wenzel, 1999), which posits that group members project their own attributes onto the prototype of a superordinate category, thereby positioning their ingroup as the most legitimate, competent, and “natural” instance of that category. In organizational contexts, this process defines the canonical ideal worker (Acker, 1990): attributes historically associated with men in dominant positions—such as continuous availability and linear careers—are recast as neutral standards of competence rather than as particular social practices.

In the GPM, prototypes are identity-anchored cultural representations enacted through evaluation practices. They occupy an intermediate space between cognitive schemas and institutional scripts, translating identity-based motives into organizational norms. Far from being fixed templates, prototypes are dynamic and power-sensitive constructs: they harden when socio-structural beliefs about legitimacy, stability, and impermeability reinforce the hierarchy, and soften when those beliefs are contested or when dominant-group members disinvest from a role.

This mechanism operates as a self-reinforcing feedback loop. Projection shapes the prototype of competence; the prototype guides evaluations and task allocations that disproportionately favor dominant-group members; visible success reinforces the perception of their superiority and prototypicality, consolidating beliefs of legitimacy and stability that sustain both the hierarchy and the projection process. Over time, these micro-level evaluations crystallize into institutional sedimentation—a normalization of dominance through the recursive triad of projection, validation, and reinforcement. What is reproduced is not merely bias, but the very standards of merit that define competence itself.

This dynamic exemplifies what Lamont (2002) describe as the boundary-making process through which symbolic distinctions become social hierarchies. In the GPM, projection operates as a boundary mechanism: it establishes who counts as a legitimate instance of a valued role and polices that boundary through evaluative practices.

This logic of prototype gatekeeping offers a unified explanation for phenomena often treated in isolation. The glass ceiling and sticky floor arise when recruitment, evaluation, and task allocation reflect a dominant-anchored standard of “merit,” confining non-prototypical actors to peripheral forms of professionalism (Acker, 1990; Kalev and Deutsch, 2018). The glass cliff appears not as progress but as strategic disinvestment: when a role becomes crisis-laden or devalued, dominant actors withdraw projection, creating precarious openings that preserve prototype authority while externalizing risk (Ryan et al., 2011). In female-typed occupations, the glass escalator results from men projecting male-coded authority onto the superordinate category, channeling sponsorship toward higher-status niches despite numerical minority (Williams, 1992, 2013).

The same mechanism illuminates reactive boundary-policing. Backlash is not a simple response to incongruity but a distinctiveness-driven defense: when dominated-group members display behaviors reserved by the prototype for the dominant, similarity cues threaten ingroup distinctiveness and elicit hostile sanction (Rudman et al., 2012; Gabarrot and Falomir-Pichastor, 2017). Tokenism amplifies these pressures, as heightened visibility renders any deviation from the prototype hyper-legible, steering tokens toward symbolic compliance roles that reaffirm the status order (Kanter, 1977). Finally, the maternal wall crystallizes the ideal-worker prototype: caregiving signals disloyalty to the ever-available standard, legitimizing downgrading even when performance is constant.

### 3.3 From evaluation to reproduction: supply–demand dynamics

The projected prototype exerts powerful effects across both sides of the labor market, linking individual evaluation, self-selection, and institutional reproduction. On the demand side, it generates a non-prototypicality penalty in evaluation. Identical behaviors are interpreted through different lenses: what appears “decisive” in a prototypical actor is read as “abrasive” in a non-prototypical one, sustaining lack-of-fit judgments (Heilman, 2001). This penalty intensifies when non-prototypical actors trespass on domains reserved for the dominant prototype, triggering a distinctiveness threat among evaluators. Their response often shifts from benevolent tolerance to hostile sanction—a reactive form of boundary-policing known as backlash (Rudman et al., 2012). For instance, women who negotiate assertively for promotions may initially be tolerated, but once their behavior too closely mirrors the dominant ideal of agency, acceptance gives way to censure. Projection thus translates dominance into everyday evaluation, embedding the attributes and life patterns of the powerful within the very standards of merit.

On the supply side, repeated exposure to these evaluative asymmetries cultivates compliance and constrains aspiration. Perceived non-prototypicality generates an internalized penalty: it reshapes goals, reduces identification with valued roles, and prompts strategic self-selection (Schmader, 2018; Guo and Schmader, 2025). As belonging uncertainty and anticipated misfit increase, effort is redirected from task engagement to self-regulation and impression management—behaviors that organizations later misread as evidence of intrinsic “preferences” or deficient motivation. Over time, these adaptive strategies produce a social reality in which the absence of non-prototypical actors from leadership tracks is interpreted not as exclusion, but as choice.

Projection, however, is not a deliberate intent to discriminate. It functions as an implicit form of ingroup favoritism, automatically activated by gender as a primary cultural frame that organizes role expectations and status beliefs (Ridgeway, 2009). Under status threat, projection intensifies: identity-defense processes mobilize to restore the dominant standard, transforming routine differentiation into reactive sanctioning. What emerges is not a series of isolated biases but a coherent mechanism through which identity-based motivation and structural opportunity converge.

The Gender Projection Model (GPM) thus unites demand and supply within a single circuit of reproduction. Dominant-anchored prototypes are inscribed into job design, evaluation metrics, and sponsorship networks, thereby structuring organizational demand for leadership and expertise. Simultaneously, repeated exposure to these same standards generates supply by shaping aspirations, effort, and anticipated costs among those perceived as non-prototypical. What appears as individual preference or “fit” is, in reality, the behavioral residue of boundary-policing and expected sanctions (Heilman, 2001; Rudman et al., 2012).

In this framework, supply is socially produced, not pre-existing. Organizational prototypes and status beliefs do not merely select from a pool of preferences—they help manufacture those preferences. The strength of this coupled process depends on socio-structural beliefs about legitimacy, stability, and permeability: when these are strong, prototypes harden and reproduction accelerates; when contested, alternative prototypes become both thinkable and actionable. The GPM therefore transforms the classical supply–demand dichotomy into a dynamic identity–structure loop, where organizational norms express dominance even as they reproduce it.

### 3.4 Boundary conditions

The explanatory scope of the GPM is delimited by the institutional and contextual conditions under which projection operates. The model applies most strongly to high-status and high-discretion roles, where evaluation is ambiguous—because criteria are discretionary, advancement depends on networked sponsorship, and gendered status beliefs remain salient. In such contexts, evaluators are more likely to default to dominant-group prototypes, amplifying projection-based exclusion.

Projection should be most pronounced when legitimacy and stability are high and boundaries appear impermeable. Under such conditions, projection functions as an invisible norm-setting force that naturalizes dominant standards. Consequently, it should weaken in settings characterized by transparent, algorithmically constrained, or collectively bargained criteria, or where counter-prototypes are institutionalized (e.g., bounded availability, team-based performance).

Yet, when legitimacy or stability is threatened, projection can also intensify in a reactive form, driven by identity-defense motives seeking to restore the threatened hierarchy (Jetten et al., 1997; Mummendey and Wenzel, 1999; Wenzel et al., 2007). In such cases, projection becomes more visible and exclusionary, translating into boundary-policing, symbolic resistance, and evaluative sanctioning. This dual logic of reflexive and reactive projection extends earlier findings in intergroup differentiation (Gabarrot and Falomir-Pichastor, 2017), situating the same identity-regulatory process at the level of organizational prototypes rather than intergroup attitudes.

While both reflexive and reactive projection serve to reproduce hierarchy, they operate through distinct motivational routes and observable manifestations. Reflexive projection is implicit, normative, and stabilizing; reactive projection is explicit, defensive, and often conflictual. The distinction is therefore not tautological but predictive: it specifies how legitimacy and stability moderate the *form, visibility*, and *targets* of projection, allowing the model to be empirically falsified.

Because prototypes are historically situated, the GPM predicts cross-field and cross-national variation tied to institutional logics and care regimes that define what “availability,” “authority,” or “competence” mean in practice.

Although the model has been elaborated for gendered fields of power, its mechanism is structurally generalizable. Projection varies with positional power rather than identity category: dominance, in this framework, is a relational and situational property, not an essence. The model therefore anticipates intersectional differentiation, with distinct projection patterns across social positions and contexts.

In sum, the GPM operates most strongly where evaluation is ambiguous, norms are discretionary, and hierarchies are perceived as legitimate and stable. When these conditions are contested, alternative prototypes—and new forms of inclusion—can emerge. These boundary conditions not only delimit the model's theoretical scope but also yield testable predictions regarding when and how projection effects should appear—developed in the next section.

## 4 Testable predictions

At the core of the Gender Projection Model is a prototype-anchoring hypothesis. In valued roles, the operative prototype will be systematically closer to the dominant group's self-described attributes than to those of dominated groups, particularly under condition of high perceived legitimacy and stability. This alignment—evidence of ingroup projection—should be visible in job descriptions and evaluation rubrics, in the traits emphasized by senior incumbents, and in the implicit standards conveyed through mentoring and sponsorship.

The stronger the dominant group's identification with a role, the more its attributes should define what counts as a “good” candidate. In that sense, the GPM offers a dynamic account of inequality reproduction that unfolds across three interrelated levels: (1) how prototypes are constructed and anchored in dominant-group identity, (2) how they shape evaluations and selection processes, and (3) how they evolve or erode under conditions of structural change.

At the perceptual level, the GPM diverges from stereotype-based or role-congruity theories by treating prototypes as *perceiver-contingent* rather than culturally shared. Dominant-group evaluators—especially those highly identified with their ingroup and the valued role—will assign greater weight to agentic and availability cues, treating these cues as defining attributes of competence. In contrast, evaluators from dominated groups will broaden or re-weight the prototype (e.g., integrating relational/collective efficacy cues), particularly when legitimacy or stability is contested or permeability is salient.

This systematic divergence in prototype content across perceivers constitutes the first diagnostic signature of the model—an empirically testable marker of projection.

At the evaluative level, these perceptual differences produce systematically asymmetric outcomes. Perceived candidate–prototype similarity should predict more favorable judgments, but the slope will be steeper for dominant-group candidates, whose similarity with the prototype is read as natural fit. In contrast, equivalent similarity for dominated-group candidates will be discounted or reinterpreted through the lens of their perceived non-prototypical category membership.

At high similarity, responses to dominated-group candidates may even turn more hostile as similarity cues can trigger a distinctiveness threat among dominant-group perceivers, shifting from paternalistic tolerance to hostile sanction (backlash). This predicted downturn—reflecting nonlinearity and group-contingent slope—differentiates the GPM from lack-of-fit and shared-stereotype models that treat similarity effects as uniform across perceivers and targets.

At the structural level, the strength of these asymmetries depends on how legitimate, stable, and impermeable the hierarchy is perceived to be. When legitimacy and stability are high, prototypes crystallize around dominant-group traits, and deviations are sanctioned. When those beliefs are contested or permeability increases, prototypes soften and diversify, allowing counter-normative traits to gain legitimacy. Interventions that reframe evaluative standards—from continuous availability to results orientation, for instance—should therefore attenuate penalties for dominated-group candidates without lowering competence thresholds, revealing the normative rather than descriptive nature of prototypes.

Over time, the same mechanism explains when and how organizational change unfolds. As a role's symbolic value erodes or crisis risk rises, dominant-group identification and projection recede; appointments of dominated-group members become more likely but under precarious conditions—a reformulation of the glass-cliff pattern centered on *dominant-group disinvestment* rather than mere “signals of change.” Symmetrically, sustained exposure to excluding prototypes raises anticipated misfit and belonging uncertainty among dominated-group members, thereby shaping aspirations, applications, and mobility decisions.

Taken together, these dynamics also define the model's empirical scope and falsifiability. The GPM conceives prototypes not as fixed cognitive templates but as identity-sensitive constructions that vary with both social identity dynamics and socio-structural beliefs. If prototypes do not vary with the identity and identification of dominant groups—or if evaluators from dominant and dominated groups generate identical prototypes regardless of legitimacy or stability cues—the model collapses into shared-stereotype accounts. Similarly, in the case of glass-cliff openings, stereotype and projection mechanisms yield opposite predictions: if increased similarity between “female” and “manager” precedes women's appointments, stereotype explanations prevail and the GPM is falsified; if decreased similarity between “male” and “manager” precedes such openings, the GPM gains support.

Thus, the same parameters that drive prototype variation—group identification and the perceived legitimacy, stability, and permeability of hierarchy—also define the conditions under which the model can be disproven. If identity-contingent shifts fail to appear, or if shared-stereotype mechanisms account for outcomes equally well, the GPM must yield to alternative frameworks. Its theoretical strength lies precisely n its vulnerability to disconfirmation—in the fact that it can, in principle, be proven wrong.

## 5 Research agenda and practical implications

### 5.1 Research agenda

Because the GPM is explicitly falsifiable, future research should also map its boundary conditions—the contexts in which projection weakens or reverses. Comparative and intersectional studies could test how the mechanism varies across occupational fields, national regimes of care, or social categories. Such analyses would clarify whether the GPM captures a general identity process or a historically bounded one, and how distinct dimensions of inequality combine or invert across institutional contexts.

The GPM offers a unifying programmatic framework for understanding the persistence of gender inequality, but its value depends on systematic empirical testing and refinement. Three research priorities follow.

First, we should develop reliable methods for mapping the operative prototypes of valued roles within organizations. These should take into account both explicit descriptors, such as job advertisements and evaluation rubrics, and the implicit normative cues conveyed through mentoring, sponsorship, and informal interactions. These measurements should be systematically linked to the group membership, identification, and socio-structural beliefs of evaluators.

Second, we should test the causal mechanisms and moderators specified by the model. Experiments and field-based quasi-experiments can manipulate legitimacy, stability, and permeability cues to examine their effects on prototype content, evaluation biases, and candidate self-selection. Such designs would make it possible to detect the predicted non-linear backlash effects that occur when non-prototypical candidates approach a dominant-anchored prototype.

Third, we should trace feedback loops between prototypes, evaluations, and socio-structural beliefs over time through longitudinal studies. Comparative analyses across sectors and national contexts could reveal how institutional logics, care regimes, and labor-market structures modulate projection processes. Integrating quantitative and qualitative approaches would connect identity-level mechanisms to institutional reproduction, clarifying where the GPM's predictions hold, attenuate, or reverse.

Finally, an intersectional perspective should examine how prototype content and penalties vary across intersections of gender, race, class, age, and parental status.

### 5.2 Practical implications

Building on this empirical agenda, the GPM also provides a diagnostic and strategic framework for organizational change. Rather than relying on generic awareness programs, interventions should target the operative prototypes that regulate access to prestige, resources, and recognition. This involves auditing prototypes to identify which attributes have been naturalized as “neutral” standards of competence, redesigning evaluation criteria to replace availability-based or exclusively agentic measures with context-relevant and performance-based indicators, and reforming governance structures so that prototype-defining committees are more diverse and sponsorship patterns less homophilous.

Structural changes—such as widening mobility channels, increasing transparency in decision-making, and institutionalizing counter-prototypes (such as bounded availability or collaborative leadership)—can further weaken boundary-policing mechanisms and make alternative competence standards credible.

Resistance to prototype change, however, is not merely cognitive but institutional and affective: it reflects vested interests, status anxieties, and the moral economy of merit. Understanding how such resistances operate is crucial for designing interventions that broaden prototypes without triggering defensive backlash.

In this sense, the GPM not only predicts where equality policies may fail but also where they might succeed—when they reshape identity investments as much as organizational procedures. By redefining prototypes and altering the socio-structural beliefs that sustain them, organizations can erode the mechanisms of symbolic closure and create the conditions for more equitable access to valued roles.

Taken together, these research directions and organizational strategies underscore the dual contribution of the Gender Projection Model: it offers both a roadmap for empirical inquiry and a compass for institutional transformation, bridging identity processes and organizational structures within a historically grounded and power-sensitive theory of social reproduction.

## 6 Conclusion

By bridging social psychology and critical sociology, the Gender Projection Model (GPM) offers an identity-grounded micro-foundation for structural analyses of inequality. In a Bourdieusian vein, ingroup projection operates as *habitus in action*: the incorporated schemes of the dominant shape perception and evaluation so as to reproduce the existing order (Bourdieu, 1998). When dominant actors impose their prototype on positions of prestige, they transform particular attributes into universal capital, enacting *symbolic violence* that naturalizes the conditions of their domination (Bourdieu, 1991). The ideal worker, far from being a neutral functional requirement, reflects the projection of dominant-group life conditions onto professionalism, thereby sustaining both visible and invisible barriers to equality.

Conceptually, the GPM integrates phenomena often treated in isolation—glass ceiling and sticky floor, glass cliff, glass escalator, backlash, tokenism, and the maternal wall—within a single process of prototype construction, projection, and boundary defense. It specifies how micro-level perceptual and motivational processes are translated, through organizational practices, into durable macro-structures, and it clarifies how these translations are amplified or dampened by socio-structural beliefs about legitimacy, stability, and permeability. Because prototypes are historically situated and power-sensitive, the framework also invites intersectional analyses that trace differentiated patterns of inclusion and exclusion produced at the intersections of multiple group memberships.

Beyond theoretical integration, the GPM provides empirically discriminant and cumulative predictions: it is falsified when prototypes remain invariant across evaluators or when stereotype-based mechanisms better account for change. This falsifiability grounds the model's claim to scientific accountability, distinguishing it from broader sociological metaphors of domination. The framework thus bridges identity, cognition, and structure through testable mechanisms that can be traced, compared, and historically contextualized.

Ultimately, the GPM resolves the false supply-demand dichotomy by demonstrating how demand manufactures supply. The normative force of dominant-anchored prototypes does not simply filter a pre-existing pool of candidates; it actively shapes the aspirations, self-evaluations, and career choices of those deemed non-prototypical. This process culminates in a powerful feedback loop: these patterned adaptations are then misinterpreted by evaluators as evidence of ‘natural' differences in motivation or preference, thereby legitimizing the very standards that produced them and closing the circle of reproduction. Altering the prototype—and the socio-structural beliefs that sustain it—is therefore not cosmetic but constitutive for transforming the observed distribution of preferences, performance, and placement.

Beyond its application to gendered hierarchies, the GPM offers a framework for analyzing how dominance operates across intersecting axes of inequality. In line with intersectional perspectives (Crenshaw, 1989; Collins, 1990), it conceives power not as additive layers of disadvantage but as interlocking systems that define which attributes and life conditions are valorized or marginalized within specific institutional fields. In this sense, projection constitutes a general logic of normalization through which particular social experiences acquire universal authority. Extending the model across racialized, classed, or colonial hierarchies would therefore help trace how distinct configurations of power generate different prototypes of competence, legitimacy, and worth.

By making visible the projection mechanisms that universalize the particular and naturalize hierarchy, the GPM resonates with decolonial critiques of modern epistemology (Mignolo, 2007): it exposes how neutrality operates as a form of domination. It thus offers both a diagnostic and a compass for rethinking the norms of competence and merit. It thus opens a space in which equality becomes not merely normatively desirable, but epistemically and institutionally attainable—an agenda that invites collective inquiry across disciplines and levels of analysis.

## Author's note

Fabrice Gabarrot is an Associate Professor of Social Psychology at Université Bourgogne Europe and a Lecturer at UniDistance Switzerland. His research examines the interplay between social identity, norms, and organizational inequality, with a focus on the micro–macro dynamics through which prototypes and status beliefs shape inclusion and exclusion in the workplace. This article develops the Gender Projection Model as part of an ongoing research program on identity-based mechanisms of workplace inequality, and was prepared for the special issue *Overcoming (in)visible Barriers: Gender, Work and Discrimination* in *Frontiers in Sociology*.

## Data Availability

The original contributions presented in the study are included in the article/supplementary material, further inquiries can be directed to the corresponding author.
